# Processes to manage analyses and publications in a phase III multicenter randomized clinical trial

**DOI:** 10.1186/1745-6215-15-159

**Published:** 2014-05-07

**Authors:** Kristin K Snow, Margaret C Bell, Anne M Stoddard, Teresa M Curto, Elizabeth C Wright, Jules L Dienstag

**Affiliations:** 1 New England Research Institutes, 9 Galen Street, Watertown, MA 02472, USA; 2 Division of Public Health Services, New Hampshire Department of Health and Human Services, Bureau of Public Health Statistics and Informatics, 129 Pleasant Street, Concord, NH 03301, USA; 3 Office of the Director, National Institute of Diabetes and Digestive and Kidney Diseases, National Institutes of Health, 2 Democracy Plaza, Bethesda, MD 20892, USA; 4 Gastrointestinal Unit, Massachusetts General Hospital, 55 Fruit Street, Boston, MA 02114, USA; 5 Department of Medicine, Harvard Medical School, 25 Shattuck Street, Boston, MA 02115, USA

**Keywords:** Publication guidelines, Publication processes, Publication management, HALT-C trial, Authorship assignment, Authorship allocation

## Abstract

**Background:**

The timely publication of findings in peer-reviewed journals is a primary goal of clinical research. In clinical trials, the processes leading to publication can be complex from choice and prioritization of analytic topics through to journal submission and revisions. As little literature exists on the publication process for multicenter trials, we describe the development, implementation, and effectiveness of such a process in a multicenter trial.

**Methods:**

The Hepatitis C Antiviral Long-Term Treatment against Cirrhosis (HALT-C) trial included a data coordinating center (DCC) and clinical centers that recruited and followed more than 1,000 patients. Publication guidelines were approved by the steering committee, and the publications committee monitored the publication process from selection of topics to publication.

**Results:**

A total of 73 manuscripts were published in 23 peer-reviewed journals. When manuscripts were closely tracked, the median time for analyses and drafting of manuscripts was 8 months. The median time for data analyses was 5 months and the median time for manuscript drafting was 3 months. The median time for publications committee review, submission, and journal acceptance was 7 months, and the median time from analytic start to journal acceptance was 18 months.

**Conclusions:**

Effective publication guidelines must be comprehensive, implemented early in a trial, and require active management by study investigators. Successful collaboration, such as in the HALT-C trial, can serve as a model for others involved in multidisciplinary and multicenter research programs.

**Trial registration:**

The HALT-C Trial was registered with clinicaltrials.gov (NCT00006164).

## Background

Well-designed clinical trials are essential to providing information needed by the medical and scientific communities in order to adapt or change treatments. Peer-reviewed scientific publications - the primary modes of communication among scientists - are an important measure of a clinical trial’s contribution to science. Hence the quality of such publications should be maximized. In a large-scale, long-term collaborative study, the potential exists for many papers to be prepared and published. For multicenter clinical trials that may involve thousands of study subjects and hundreds of investigators worldwide, timely publication of research findings can be complex and challenging, from choice and prioritization of analytic topics through to journal submission and revisions [1,2]. Therefore the recommendation has been made that large study groups prospectively develop a formal policy specifying authorship guidelines to avoid unpleasant surprises and controversies during manuscript preparation [3,4]. There are few scientific publications detailing strategies to promote publication timeliness in collaborative studies and no publications on planning multiple publications of inter-related data collected from a single population of study patients [5-8]. In large-group multidisciplinary health sciences research, authorship allocation based on predefined principles ensures appropriate acknowledgment for individual responsibility and contribution to a publication, necessitating guidelines for planning authorship assignments and publications [9].

The Hepatitis C Antiviral Long-Term Treatment against Cirrhosis (HALT-C) trial was a National Institutes of Health (NIH) sponsored Phase III randomized controlled trial spanning a 14-year time period. The HALT-C trial Group was comprised of 290 study-affiliated investigators, collaborators, and staff members. The collection of an enormous amount of data, and interest in using the data for secondary analyses, necessitated the establishment of a system for publication planning and management. The HALT-C trial group developed processes to guide collaborators through topic selection, equitable assignment of authorship duties and authorship order, analysis prioritization, and eventual publication of study data. The procedural approach allowed a transparent process that collaborating researchers accepted readily. Through open, proactive, and frequent communication of current and planned publications, the HALT-C trial group sought to preemptively limit conflicts, avoid bottlenecks, and reduce tension among colleagues. Herein, we describe the HALT-C trial publication processes, which may prove a useful model for scientists and administrators faced with data collection, analysis, and manuscript-prioritization decisions in other complex studies with large databases of interrelated data on one population of study patients.

## Methods

### Background

The HALT-C trial was sponsored by the National Institute of Diabetes and Digestive and Kidney Diseases (NIDDK), NIH. Initial study funding began in November 1999, with 10 clinical center sites (CCs) contracted across the United States. Separately, NIDDK funded a central specimen repository (SeraCare Life Sciences, Gaithersburg, Maryland, United States), a central virology laboratory (at the University of Washington, Seattle, Washington, United States), and a data coordinating center (DCC) (at New England Research Institutes, Watertown, Massachusetts, United States). Recruitment began in study year 1 (2000) and ended in study year 5 (2004). Patient follow-up for the randomized phase ended in study year 8 (2007), but patients continued to be followed until the end of study year 9 (2008). CC funding ended in study year 12 (2011) and DCC funding ended in study year 14 (2013).

The study investigators established several committees of which two are relevant to the current paper. A steering committee, whose membership consisted of a single lead investigator from each of the 13 independent sites (10 CCs, the virology laboratory, the DCC, and NIDDK project office), was responsible for study oversight including management of sub-committees. The publications committee maintained oversight of publications and presentations and was comprised of a representative from each of the sites.

The design of the HALT-C main trial has been previously described [10,11]. The aim of the trial was to determine whether long-term peginterferon therapy could reduce the progression of advanced chronic hepatitis C (CHC) in previous non-responders to interferon-based therapy and whether the anticipated benefits would justify the risks, inconvenience, and expense involved.

HALT-C had two major treatment phases and an observational phase. A lead-in phase used full dose peginterferon alfa-2a and ribavirin to attempt to achieve sustained virological response (SVR) among patients with advanced liver disease (defined as an Ishak fibrosis score of 3 or greater upon liver biopsy) who had previously been treated with standard interferon with or without ribavirin. Patients who did not achieve SVR were eligible for the randomized phase; a controlled clinical trial of peginterferon alfa-2a at a dosage of 90 μg per week for 3.5 years, as compared with no treatment. The primary endpoint was progression of liver disease as indicated by death, hepatocellular carcinoma, hepatic decompensation, or, for those with bridging fibrosis at baseline, an increase in the Ishak fibrosis score of 2 or more points. The difference between the treated and untreated groups in the rates of the primary endpoint was not statistically significant (hazard ratio - 1.01; 95% CI = 0.81 to 1.26, *P* = 0.91) [11]. Patients continued to be followed in the observational phase for clinical outcomes off therapy for as long as five additional years. The study was approved by the institutional review boards of each participating institution (Additional file 1) and registered with ClinicalTrials.gov (NCT00006164). All study patients provided written informed consent for participation in the trial.

In addition to the main trial, 41 ancillary studies were designed and conducted during which additional data and/or biological samples were collected and analyzed. Some ancillary studies included all enrolled trial patients, while others were conducted at selected CCs or involved a subset of patients. For the HALT-C main trial and ancillary studies, 146 different case report forms were developed and deployed over the course of the research period. A total of 33,072 patient visits and 371,684 individual case report forms were entered into the central database. In addition, 400,848 specimen samples were collected by investigators from trial subjects for storage by the central repository or for testing at the central virology laboratory. All collected data were made available for analyses.

### Publication goals

The overarching publication goal of the HALT-C trial was to disseminate the key study results to the scientific community in a timely fashion. With this goal in mind, the steering committee and publications committee had to address the concerns of the equitable assignment of authorship across multiple investigators, the best practices for selection of analyses and publication topics, the order and prioritization of conducting statistical analyses by the DCC, and the promotion of successful, collegial collaboration.

The DCC was tasked with managing the coordination of publication processes, effective facilitation of writing group interactions, determination of analysis prioritization (with investigator approval), completion of all desired analyses and manuscripts in a timely fashion, maintenance of data, analytic, and publication quality, and helping the entire study team to collaborate successfully.

### Publication guidelines

The publications committee chair used guidelines from older NIH studies to develop the HALT-C publication and presentation guidelines. After review by publications committee members, version 1 of the guidelines was initially approved by the steering committee in study year 1. The guidelines underwent two amendments in study year 2 and 3, which added authorship guidelines for non-HALT-C investigators (version 2) and required inclusion of a financial disclosure paragraph in all manuscripts (version 3), along with other minor clarifications (see Additional file 2 for the final version of the guidelines). The guidelines provided the basis and reference document to support the publication of HALT-C trial research results and established standard procedures for authorship. The guidelines were designed to achieve six goals. First, to promote the timely and high-quality presentation and publication of findings. Second, to support broad and equitable participation by HALT-C trial investigators in presentations and publications. Third, to prospectively define a set of equitable rules and guidelines to determine authorship and the order in which authors are listed. Fourth, to select topics for publication and presentation, assign authors to writing groups, and set priorities for publications and presentations. Fifth, to provide editorial support and timely review for presentations and publications. Sixth, to defend the academic freedom of HALT-C trial investigators to publish results emanating from the trial while providing limitations on the premature dissemination of results that could threaten the integrity of collective data and jeopardize publication in high-impact journals.

## Results

The HALT-C trial group developed organizational procedures and processes to facilitate analyses and publications during the course of the trial. First, the actions of the publications committee during the study are described followed by information on resulting publications.

### Publications committee actions

#### Writing groups

Manuscript writing was assigned to a writing group consisting of three to four investigators, one of whom was designated the writing group Chair and responsible author, as well as a DCC statistician. The publications committee nominated the writing group members which was followed by approval from the steering committee. The publications committee Chair ensured equitable assignments to writing groups over the course of the study. In the opinion of the authors, limiting each writing group to a maximum of four investigators was a key factor in the efficiency of the writing groups.

#### Manuscript concept sheets

The publications committee planned a ‘main outcome’ paper addressing the primary objective of the HALT-C trial: the impact of long-term antiviral therapy on pre-cirrhotic and cirrhotic CHC [11]. For other papers, the publications committee asked investigators to suggest topics by submitting manuscript concept sheets (MCSs) to the publications committee. The DCC developed a two-page MCS template which included a brief description of the background, hypothesis and purpose, a definition of the subjects to be included, a list of variables of interest, and planned statistical analyses. In practice an investigator drafted the MCS with statistical input from the DCC. The publications committee judged each MCS on scientific merit, availability of appropriate data, and ability of the DCC to accommodate the analyses. If an overlap in content existed between two proposals, a consolidated MCS was developed with oversight by the Publications Committee. Interested collaborators were solicited to form the proposed writing group. Writing group membership could be altered or augmented by the publications committee.

At the peak of publications planning during study year 7, 60 MCSs were submitted and reviewed at an in-person steering committee meeting. After consolidation of some topics and removal of other topics that had insufficient data or limited scientific interest, 40 MCSs were approved by consensus. Investigators were then asked to indicate their top four choices of interest. The publications committee Chair then assigned investigators to manuscripts, balancing investigators across the CCs, with subsequent approval of these assignments by the steering committee. Use of the MCS process reduced overlap between and among manuscripts and provided a means for equitable authorship assignment.

#### Prioritization of topics and analyses

The publications *c*ommittee categorized MCSs into the three manuscript categories: (1) Study-wide main papers that represented reports of the main outcomes of the trial, based on analysis of study-wide data collected by all CCs. (2) Secondary papers that addressed issues more peripheral to the main study outcomes but that were based on data collected as part of the main study at all CCs. This category included ancillary studies that were conducted at all CCs. (3) Local papers that represent reports of data collected from locally initiated and separately conducted ancillary studies unique to one or several CCs.

Within each of the three categories, MCSs were further prioritized into high, medium, and low levels based on importance of the topic. The steering committee initially approved priority levels at an in-person meeting. Priority levels were reviewed regularly and were subject to revision by the steering committee. For each new analysis the DCC started the analysis in order of the assigned priority level (from highest priority to lowest).

The DCC provided information on the number of analyses that could be conducted concurrently based on the capacity for support by DCC staff and statisticians, and whether all approved analyses could be completed within the timeframe of study funding. The DCC forecasted the capacity for conducting analyses based on annual contract funding and staffing levels, with each statistician assigned to between two and four analyses at a time.

**Figure 1:**
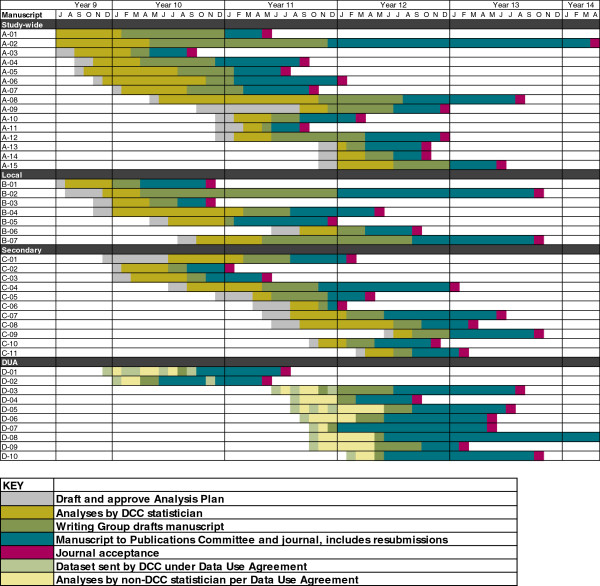
**HALT-C manuscript timelines.** This figure includes publications that were tracked starting in July of study year 9. Thirty-three publications, classified as study-wide, local, or secondary, were analyzed at the DCC and ten publications were analyzed at other institutions using a DUA. The color codes indicate the activity stage. DCC, data coordinating center; DUA, data use agreement.

Further, the publications committee solicited feedback from the DCC when papers were taking more analytic time than anticipated, or when writing groups were slow to complete milestones. During study year 7 statistical requests and workload increased because final study results were expected in study year 8. In response, the DCC developed a manuscript timelines spreadsheet that listed all ongoing analyses being conducted, with information on stage of analysis (Figure 1). The spreadsheet was updated and distributed monthly to the publications committee and steering committee, which allowed for the easier review and forecasting of upcoming work. This process was effective in forecasting DCC capacity and resource allocation within budget and staffing constraints.

#### Analysis and writing processes

The publications committee agreed that all papers should present a coherent message, therefore, a list of approved study outcome definitions was developed with input from the DCC statisticians. The primary and secondary outcomes of the randomized trial were defined in the protocol and the statistical analysis plan, and all other analyses for any publication were required to use the approved outcomes. Some publications used alternate outcome definitions that had not been included in the protocol. Alternate outcome definitions were proposed by writing groups, reviewed by the DCC, and then required publications committee approval. As the study follow-up time was extended, additional outcome definitions were added to define the time intervals for survival analyses. The publications committee informed ongoing writing groups when new outcome definitions were approved for use.

When a MCS came up in the priority-based queue, a standard 14-step process was followed by the writing group and assigned DCC statistician: (1) The statistician drafted a detailed analysis plan (AP), which included descriptions of the cases to be included, planned variables, analytic approaches, and mock tables; (2) The writing group and statistician reviewed, revised, and approved the AP, with the understanding that the group had to adhere to the AP; (3) The statistician performed analyses according to the AP, soliciting help from other statisticians as needed; (4) The statistician prepared summaries of the results for discussion by the writing group; (5) Writing group conference calls were scheduled at least once per month to review analytic results and tables. Writing group sessions were also built into the agendas of semiannual in-person steering committee meetings. Regular attendance and active participation in meetings and teleconferences were expected; (6) The writing group Chair directed the analyses of each manuscript. He or she worked one-on-one with the statistician, and all analysis requests went through the Chair; (7) The writing group Chair directed the writing of each manuscript. He or she prepared action items, assigned writing and reviewer tasks, and was responsible for overseeing the completion of tasks on schedule. The statistician assisted with drafting assigned sections of the manuscript and preparing all tables and figures; (8) The DCC assisted the writing group Chair with scheduling calls and meetings. The DCC sent reminders two to three days prior to each meeting, along with all associated materials to be reviewed; (9) The DCC held weekly statistical team meetings to review timelines, facilitate cross discussion of related analyses, and offer help with writing group interactions; (10) A second DCC statistician performed quality control review of analyses to ensure accuracy of programming and data presented in the final manuscript; (11) The writing group Chair reported on the current status of manuscript preparation at monthly publications committee and steering committee calls. If progress was stalled, committee members discussed options and workarounds, including the possibility of the replacement of writing group members or a change in the analysis priority level; (12) The writing group Chair finalized the manuscript with input from all co-authors and the DCC forwarded the manuscript to the publications committee and NIDDK for review; (13) The writing group Chair was responsible for addressing internal reviewer comments and submitting the manuscript to the chosen journal; (14) The writing group Chair was responsible for responding to any journal editor and outside reviewer comments and for resubmission. The statistician performed re-analyses as needed. Analyses of manuscripts being revised for resubmission received high priority.

#### Data use agreements

In study year 7, the steering committee agreed to allow the sharing of analytic datasets with approved investigators which permitted the generation of additional manuscripts, especially in topics and disciplines requiring specialty statistical input (such as Genome Wide Association Studies). The steering committee developed a data use agreement (DUA) template, which was completed by interested investigators (Additional file 3). A DUA was reviewed by the DCC to determine whether requested data were available and to estimate the amount of time required to produce the requested dataset. The steering committee and NIDDK then approved the DUA. After a dataset was provided, analyses were conducted by a non-DCC statistician. A DCC statistician performed a statistical quality control check of the drafted manuscript. The resulting manuscript underwent publications committee review following the standard process.

#### Authorship attribution

The decision-making process for authorship attribution and order of authors was outlined *a priori* in the publication guidelines. The guidelines contained the clear statement that ‘Authors should participate in the writing of the paper according to guidelines of the International Committee of Medical Journal Editors’ (ICMJE) [12]. The ICMJE guidelines stipulate that authorship requires involvement in planning, analysis, or writing of the manuscript, contribution to concept, design, and analysis, a role in the drafting of the article and/or revising it critically for important intellectual content, and final approval of the version to be published. The writing group Chair controlled task allocation in writing the paper and acted as a gatekeeper in the attribution of authorship and authorship order, based on the division of labor during the writing of the paper. Each manuscript’s statistician was included as an author. Additional authors were listed in order of the number of patients enrolled at the corresponding site, in adherence to publications committee guidelines, which also allowed for the designation of a different author order based on exceptional effort and input during manuscript preparation. The publications committee Chair was also consulted if necessary.

#### Editorial processes

Each fully drafted manuscript was submitted to the publications committee and NIDDK for review. Two publications committee members who were not members of the writing group for the paper were designated to provide a timely in-depth review for editorial clarity and data integrity. All modifications requested by the publications committee members or in-depth reviewers were sent to the writing group Chair to address. The manuscript was submitted for publication after obtaining approval from all authors, the publications committee, and NIDDK.

#### Administrative processes

To help the writing group Chair with the administrative details of manuscript submission, the DCC developed the supplementary sections for every paper. These sections contained each author’s name, degree, institutional affiliation, acknowledgments of study funding sources, acknowledgments of appropriate study personnel, and financial disclosures paragraphs.

To protect the publication process from a real or apparent conflict of interest, all investigators, co-investigators, and manuscript co-authors were required to disclose all relationships with pharmaceutical companies or other relevant entities. The DCC coordinated this annual disclosure process and maintained a log of all potential conflicts of interests. The DCC used this log to draft a financial disclosure section for each manuscript. Each co-author was required to review and confirm that the disclosure information was up-to-date for each manuscript.

Contributors who did not meet the criteria for authorship, but nevertheless contributed to the development of the research or manuscript, were listed in the acknowledgments section of each paper. The list of contributors was maintained by the DCC. Each principal investigator was responsible for reviewing and approving the inclusion of acknowledged persons from their CC on every paper.

The supplemental sections were distributed for review and approval prior to their insertion into the final manuscript for journal submission. The DCC collaborated with the writing group Chair to ensure that these sections were accurate in the submission version of the manuscript.

#### HALT-C publications

The publications committee assigned a writing group to develop the HALT-C trial design manuscript in study year 3 [10]. The paper provided detailed information on study design, hypotheses, outcomes, inclusion and exclusion criteria, sample size and power calculations, study execution, and numbers of patients screened, randomized, and followed. Publication of this paper early in the study cycle provided a citation for use in all subsequent papers about basic information on study design.

The HALT-C trial main outcome manuscript received top analytic priority and was produced efficiently, following the processes described above [11]. Introductory sections were drafted and tables predefined by the writing group. An analytic plan for statistical programming was adopted that allowed rapid data analyses after data lock occurred in study year 8. The manuscript was written, reviewed, and approved quickly; and submitted and accepted by a leading journal nine months later, despite the null results of the primary outcome. During the time between initial submission and acceptance by the journal, the writing group responded to three requests for additional analyses or revisions by reviewers. The paper was published five months after acceptance.

**Figure 2:**
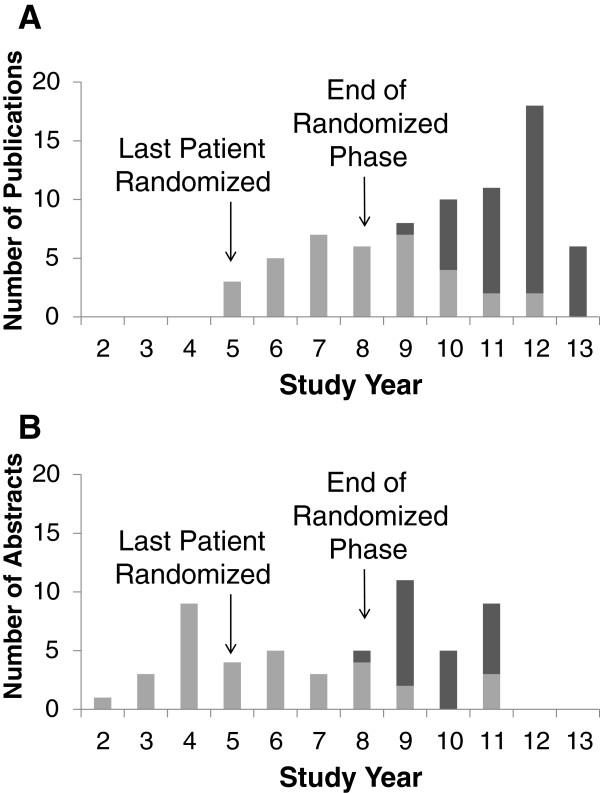
**Number of HALT-C publications and abstracts by year published. A**: The light grey portions are for the 36 publications that used only baseline or lead-in phase data and the dark grey portions are for the 38 publications using data from the randomized phase of the HALT-C trial. **B**: The light grey portions are for the 33 abstracts that used only baseline or lead-in phase data and the dark grey portions are for the 22 abstracts using data from the randomized phase of the HALT-C trial. Study year represents the number of years from the start of the study.

Including the design and main outcome papers, 73 papers and one letter were published in 23 peer-reviewed journals across the fields of general medicine, gastroenterology and hepatology, psychiatry, virology, and clinical trials (Table 1) [10,11,13-78]. A total of 28 of the 74 publications were study-wide main papers that represented main outcomes of the trial, 25 were secondary papers, and 21 were local papers. A total of 39 (53%) of these papers were published during a three-year period after the randomized trial was completed (Figure 2A). A total of 64 of the 74 publications were analyzed by the DCC. The remaining 10 were prepared under a DUA but were considered important to the study and were tracked by the publications committee. One manuscript prepared under a DUA and tracked by the publications committee was rejected by one journal and has not been submitted to another journal.

**Table 1:** HALT-C publications by journal

**Journal names**	**Number of publications**	**Number of unique first authors**	**2012 impact factor**
1. *New England Journal of Medicine*	1	1	53.30
2. *Gastroenterology*	14	10	11.68
3. *Hepatology*	21	14	11.67
4. *Gut*	1	1	10.11
5. *Journal of Hepatology*	4	4	9.26
6. *American Journal of Gastroenterology*	6	3	7.28
7. *Journal of Infectious Diseases*	1	1	6.41
8. *Clinical Gastroenterology Hepatology*	4	4	5.63
9. *Gastrointestinal Endoscopy*	1	1	4.88
10. *Journal of Clinical Microbiology*	1	1	4.15
11. *PLoS One*	3	3	4.09
12. *Journal of Viral Hepatitis*	2	2	4.09
13. *Liver International*	1	1	3.82
14. *Alimentary Pharmacology Therapeutics*	5	3	3.77
15. J *Affect Disorders*	1	1	3.52
16. *Pharmacogenetics and Genomics*	1	1	3.49
17. *Journal of International Neuropsychology Society*	1	1	2.76
18. *Clinical Trials*	1	1	2.36
19. *Virology Journal*	1	1	2.34
20. *Journal of Clinical Experimental Neuropsychology*	1	1	2.13
21. *Psychosomatics*	1	1	2.12
22. *Digestive Disease Sciences*	1	1	2.12
23. *Contemporary Clinical Trials*	1	1	1.81
**TOTAL**	**74**		

Of the 74 publications, 35 different investigators were first authors. First authors came from all 13 sites that were represented on the steering committee, a testament to the intellectual engagement from all centers in the study. A total of 123 investigators were authors at least once among the total of 776 co-authors listed on all publications. Co-authors included members of the manuscript’s writing group and others who had contributed to the publication, as specified in the guidelines.

The publications guidelines also described procedures for the submission of abstracts to meetings of professional societies. Proposals for abstracts had to be submitted to the publications committee six weeks before the submission deadline so that the DCC could determine the workload. Drafts of the abstract had to be submitted to the publications committee seven days before this deadline. Between study year 1 and study year 11, 55 abstracts were accepted for presentation at annual meetings (Figure 2B). Most of these abstracts were linked to manuscript proposals and all were eventually included in a publication. The median time from abstract presentation to publication was 1.5 years, range 0.25 to 4.75 years.

**Figure 3:**
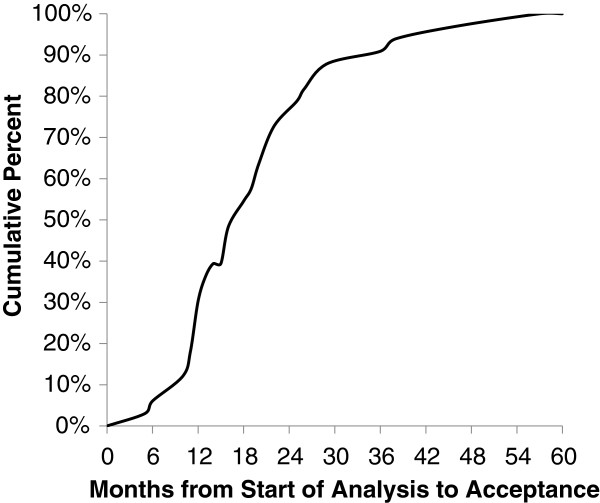
**Cumulative distribution of time from start of analysis to journal acceptance.** These 33 HALT-C publications, closely tracked by the publications committee, had a median time from start of analysis to acceptance of 18 months (range 5 to 57).

The DCC tracked progress of manuscript proposals during the most active period of analysis and publications when more than two dozen proposals were planned (study years 9 to 13) (Figure 1). During this timeframe, the equivalent of four full time biostatisticians contributed to the publication effort. A total of 33 proposals were initiated, analysis plans were prepared, statistical analysis by a DCC statistician conducted, and a manuscript draft prepared for publications committee review. The median time for these steps was 8 months (range 3 to 28 months). The bulk of this time was taken up by data analysis (median 5.0 months; range 1 to 17 months) and manuscript preparation by the writing group (median 3.0 months; range 1 to 21 months). Time from submission to the publications committee to acceptance by a journal ranged from 1 to 33 months (median 7 months), and the median total time from analysis to acceptance was 18 months (range 5 to 57). Figure 3 shows the cumulative distribution of the time from the start of analysis to acceptance by a journal for these 33 publications. The first 32 publications were not included in either Figure 1 or Figure 3 because these publications were being prepared during a time when the analysis workload was relatively light and the DCC did not track their progress. One of these 32 was the primary results manuscript, which was not included because of the constraints imposed by having to wait for the final data lock to complete the analyses.

During the course of the trial, investigators of other NIH-sponsored multicenter trials requested access to the HALT-C trial publication and presentation guidelines. These requests were submitted to the publications committee, which approved sharing of the document as long as attribution to the HALT-C trial was disclosed. The guidelines document is included as Additional file 2, and may be used by other trials with attribution.

## Discussion

Effective publication guidelines must be comprehensive, must be implemented early in a trial, and require active management by study investigators. The HALT-C trial goals were met through developing and following publications processes, with collaboration and compromise by collaborators. Paramount in this detailed process was the high priority placed on anticipation and preemption of potential publication problems and conflicts. Disagreements among investigators over authorship participation and order were avoided by adhering to the approved publication guidelines.

Despite our best efforts, the processes did not always work optimally. Some manuscripts took longer to produce than anticipated, resulting from delays at the levels of analysis, writing, review, or submission. In two instances, the publications committee replaced a writing group Chair when the writing process lagged. The DCC could not always accommodate requests for analyses in order to meet conference deadlines for abstract submissions as analyses for higher priority manuscripts had precedence. Similarly, the DCC could not accommodate requests for lower level priority MCSs when all statistician effort was committed to higher priority MCSs. If investigators expressed impatience with timing of analyses, the DCC could request a review of priorities and assistance in resolving disputes from the steering committee or the publications committee.

Our analysis of the time interval from the start of analysis to journal acceptance included only the last 33 manuscripts prepared by the DCC statisticians. This time interval would likely have been somewhat longer if we had included the earlier manuscripts that were prepared at a time when there was less pressure for timely publication.

In spite of the large scope, 14-year duration, and complexity of the trial, the HALT-C trial group developed guidelines and processes that allowed the group to assign authorship equitably, select publication topics collaboratively, determine priorities for analyses, facilitate writing group activities, and promote a high degree of productivity, culminating in the publication of 73 papers and one letter (and the supporting abstracts and presentations prepared during their development). All HALT-C results presented in abstract form were subsequently published in full, a rate far higher than that found in other studies [79,80].

The processes that proved most effective at accomplishing the publication goals were the following: requirement for formal MCSs and APs, assignment of small, focused writing groups that adhered to approved outcome definitions, analytic prioritization assigned by the publications committee and tracked on a manuscript timelines spreadsheet, and tight control of publications committee reviews and manuscript approvals prior to journal submission.

The HALT-C trial publication processes allowed for effective dissemination of study results to be published in a broad range of high-quality journals in a timely fashion [5,7,8,79,81]. The findings of the HALT-C trial have had important implications for the treatment of patients with treatment-resistant, histologically advanced chronic hepatitis [11].

Further, study results were published in other scientific areas beyond the field of gastroenterology, including microbiology, neuropsychology, and pharmacogenomics. The number and quality of manuscripts published justified the funding and effort devoted to the study over 14 years. The rich HALT-C trial database, as well as the remaining biological specimens and genetic data, have been submitted to the NIDDK Repository and are available for other researchers to investigate and query [82]. The HALT-C trial should continue to be a valuable source of scientific data and biospecimens for future investigation and years to come [83].

## Conclusions

Group, team, collaborative, or network studies are becoming more frequent in health-sciences research. Good coordination and planning are necessary to assure timely, complete, and accurate reporting of results to the scientific community, as well as to avoid tensions and conflicts that can arise in the context of analysis of a large collaborative research study. Adherence to our organized publication processes led to success in achieving these goals. Although not planned to be a generalizable system, our example of how such collaboration was achieved successfully in the HALT-C trial can be useful as a model for others involved in or planning multidisciplinary and multicenter research programs.

## Abbreviations

AP: Analysis plan; CC: Clinical center; CHC: chronic hepatitis C; DCC: Data coordinating center; DUA: Data use agreement; HALT-C: Hepatitis C antiviral long-term treatment against cirrhosis; ICMJE: International Committee of Medical Journal Editors; MCS: Manuscript concept sheet; NIDDK: National Institute of Diabetes and Digestive and Kidney Diseases; NIH: National Institutes of Health; SVR: sustained virological response.

## Competing interests

The authors declare that they have no competing interests.

## Authors’ contributions

KS conceived of the study, participated in the design of the study, helped with acquisition and interpretation of the data, and drafted and revised the manuscript. MB participated in acquisition and interpretation of the data and helped draft and revise the manuscript. AS and TC participated in acquisition and interpretation of the data and helped draft and revise the manuscript. EW participated in acquisition and interpretation of the data, performed the statistical analysis, and helped draft and revise the manuscript. JD participated in interpretation of the data and helped draft and revise the manuscript. All authors read and approved the final manuscript.

## Authors’ information

Kristin K Snow was an employee of New England Research Institutes during this study and is now an employee of the New Hampshire Department of Health and Human Services, Concord, NH.

## Supplementary Material

Additional file 1 **Names of Institutional Review Boards that approved HALT-C.** List of IRBs that approved HALT-C. Click here for file

Additional file 2 **HALT-C Trial Publication and Presentation Guidelines.** Publication and Presentation Guidelines. Click here for file

Additional file 3 **HALT-C Trial Data Use Agreement.** Data Use Agreement template. Click here for file
