# Supplementary material for: Processes to manage analyses and publications in a phase III multicenter randomized clinical trial
Source: Trials. 2014 May 7;15:159. doi: 10.1186/1745-6215-15-159 (PMC4040510; doi:10.1186/1745-6215-15-159)
Supplement: Additional file 1 — Names of Institutional Review Boards that approved HALT-C. List of IRBs that approved HALT-C. [file 1745-6215-15-159-S1.doc]

***H****epatitis* ***C******A****ntiviral* ***L****ong-term* ***T****reatment against* ***C****irrhosis*

**HALT-C Trial**

# Names of Institutional Review Boards that approved HALT-C

#

# Human Subjects/IRB

# University of Massachusetts Medical School

# Worcester, MA

# Human Subjects Protection Office

# University of Connecticut Health Center

# Farmington, CT

# Biomedical IRB

# Saint Louis University

# Saint Louis, MO

# Partners Human Research Committee

# Boston, MA

# Colorado Multiple Institutional Review Board

# Aurora, CO

# University of California, Irvine Institutional Review Board

# Irvine, CA

# IRB (Subcommittee on Human Studies)

# Long Beach VAMC Research Health Care Group

# Long Beach, CA

# Institutional Review Board

# The University of Texas Southwestern Medical Center

# Dallas, TX

# Institutional Review Board

# University of Southern California Health Sciences Campus

# Los Angeles, CA

# Institutional Review Board for Human Subject Research

# University of Michigan Medical School

# Ann Arbor, MI

# Office of Research Subjects Protection

# Virginia Commonwealth University

# Richmond, VA

# Institutional Review Board

# National Institute of Diabetes and Digestive and Kidney Diseases

# National Institutes of Health

# Bethesda, MD

# Institutional Review Board

# New England Research Institutes

# Watertown, MA
