# Supplementary material for: Processes to manage analyses and publications in a phase III multicenter randomized clinical trial
Source: Trials. 2014 May 7;15:159. doi: 10.1186/1745-6215-15-159 (PMC4040510; doi:10.1186/1745-6215-15-159)
Supplement: Additional file 2 — HALT-C Trial Publication and Presentation Guidelines. Publication and Presentation Guidelines. [file 1745-6215-15-159-S2.pdf]

Version 3.3.2 (3/9/06)

HALT-C Trial Publication and Presentation Guidelines

A. GOALS

1. Promote timely and high-quality presentation and publication of findings.

2. Support broad and equitable participation by HALT-C investigators in presentations and publications

3. Define prospectively a set of equitable rules and guidelines that can be used to determine authorship and the order in which authors are listed

4. Select topics for publication and presentation, assign authors to Writing Groups, and set priorities for publications and presentations

5. Provide editorial support and timely review for presentations and publications

6. Defend the academic freedom of HALT-C investigators collectively to publish results emanating from the Trial while providing limitations on publication of results from any one Center that could threaten the integrity of collective data

B. SCOPE OF GUIDELINES

1. These guidelines will apply to original manuscripts (including methodology, validation, laboratory approaches, etc.), abstracts, oral and poster presentations, letters to the editor, meeting proceedings, extended abstracts that include data collected as part of the HALT-C Trial, and reviews that include original HALT-C Trial data not previously published in a primary source. In addition, these policies apply to both the results of the Clinical Trial and to the results of Ancillary Studies related to the Trial.

2. These policies remain in effect even after formal conduct and funding of the Trial are complete.

3. For purposes of publications and presentations, all data derived from the HALT-C Trial or from specimens collected during the Trial are the collective intellectual property of the study investigators, not those of any individual investigator, collaborating investigator, or the study sponsors from government and industry. (Legally, the data and specimens collected are the property of the US Government.)

4. These guidelines will become part of the Policy Manual for the Trial.

C. PUBLICATIONS COMMITTEE

1. The Publications Committee of the HALT-C Trial will consist of the Principal Investigator — or his/her designee — from each of the 10 Clinical Centers, the Data Coordinating Center, the Virology Center, and the NIDDK.

2. The Chairperson of the Publications Committee will be elected for a term of one year by members of the Publications Committee. During the month of April, and no later than May 1 of each year of the Trial, the election will be coordinated by the Data Coordinating Center. The number of consecutive or interrupted terms that a chairperson may serve will not be limited.

3. The Publications Committee will mediate and settle all disputes and conflicts over publications issues, priorities, and authorship, etc., among study investigators. Investigators who perceive inequities in authorship or other problems relating to authorship should discuss these concerns with the Publications Committee chairperson; if the difficulty cannot be settled in this informal manner, the concerned investigator should submit a letter to the Publications Committee chairperson outlining the problem. The document will be reviewed and discussed by the Publications Committee, and a formal written reply will be made to the investigator. If the chairperson of the Publications Committee has a perceived or real personal stake in the outcome of the conflict and cannot preside in an unbiased way, he or she should be recused, and another member of the Publications Committee should assume the chairperson's role in resolution of the dispute. If Publications Committee deliberations fail to resolve such a dispute, the dispute will be submitted for resolution to the Steering Committee.

4. The Publications Committee reserves the right to amend Publications Committee Guidelines as necessary, in order to clarify their intent.

D. TYPES OF PUBLICATIONS

1. Study-Wide, Main Papers that represent reports of the main outcomes of the Trial, based on analysis of study-wide data

2. Secondary Manuscripts that address issues more peripheral to the main study outcome but that are based on data collected as part of the main study. This would include ancillary studies that are study-site-wide.

3. Local Papers that represent reports of data collected from locally initiated and separately conducted ancillary studies unique to one or several sites

4. Methodology/Validation Papers

5. Abstracts, meeting proceedings, extended abstracts, oral and poster presentations

6. Letters to the Editor

7. Press Releases

E. AUTHORSHIP

1. Authors should participate in the writing of the paper according to guidelines of the International Committee of Medical Journal Editors (N Engl. J Ed 1991; 324:424-8). Those who participated in <u>conception and design, analysis and interpretation of data, drafting of the manuscript, critical revision of the manuscript relating to important intellectual content, and final approval of the manuscript</u> should be included as authors. Statistical expertise and virology expertise that relate directly to the conduct of the study are additional criteria for authorship. Provision of study material or patients; collection and assembly of data; provision of administrative, technical, or logistic support; and obtaining funding do not necessarily merit authorship but should be considered on a case-by-case basis, especially when other contributions are included. Honorary authorship will not be considered.

2. For each Study-Wide Main Paper, the writing of the manuscript will be assigned to a Writing Group, to consist of three to four Trial Investigators, one of whom will be designated the Chairperson or Responsible author, plus a representative of the Data Coordinating Center. The Publications Committee will nominate the Writing Group and Chairperson and send the selection to the Steering Committee for final approval. The Writing Group, plus any ad hoc contributors who fulfill criteria for authorship, will be included as authors. The Writing Group for each publication will be listed in an Acknowledgments section of the manuscript.

3. Selection of first authors: Unless he/she delegates otherwise, the Chairperson of the Writing Group will be the first author.

4. For Study-Wide Main Papers, the list of authors should be limited to one investigator per Clinical Center, Virology Center, and Data Coordinating Center, as well as the NIDDK. Because so many personnel from each Center are participating in the Trial, including all personnel is impractical and contrary to journal guidelines and limitations. The selected list of masthead authors will end in “and the HALT-C Trial Group.” All study participants should be listed in an appendix or footnote that identifies study centers and the roles of participants per center. For journals that limit the number of masthead authors, the task of designating a smaller number of masthead authors will fall to the Publications Committee. In case this contingency arises, the following order of authorship will apply until the journal’s limit is reached:

    a. The Writing Group

   b. Investigators felt by the Writing Group to have made special contributions to the concept, design, or analysis of the study (often these will already be included in the Writing Group)

   c. Investigators felt by the Writing Group to have contributed special effort to the execution of the study (often these will already be included in the Writing Group)

   d. Investigators ranked by number of study subjects enrolled.

5. Order of Authorship: For some manuscripts, the contribution of primary investigators may be equivalent among study Centers. For others, especially secondary manuscripts, manuscripts describing ancillary studies, or manuscripts suggested by a subset of study investigators, level of input will be considered in order of authorship. For all manuscripts, factors to be included in decisions about order of authorship are contribution to concept, design, and analysis; role in drafting of the article and/or revising it critically for important intellectual content; number of study patients enrolled; completeness/integrity of the data and specimens from the investigator's site; and leadership role. The Publications Committee may amend the order of authorship to recognize an exceptional contribution to the study or the manuscript by an individual Investigator. The Writing Group for each Main Paper will suggest an order of authorship that will be approved first by the Publications Committee and then the Steering Committee.

6. The Publications Committee will oversee the writing and authorship of manuscripts and insure that the tasks of writing and the recognition of authorship are distributed fairly among study investigators and not dominated by any one investigator or study center.

7. Failure to complete assignment:

   a. If a Writing Group does not complete its work on a manuscript or fails to meet timeline milestones, the Chairperson of the Publications Committee may reassign the roles of first author and/or select new Writing Group members. This exigency will be exercised if no draft is produced within 4 months of the availability of a clean data set for the manuscript.

8. Investigators who have left HALT-C Trial

   a. Over the multi-year course of the HALT-C Trial, one or more Investigators or Co-Investigators are likely to leave their institutions, change primary interest, or terminate their roles in the HALT-C Trial for a variety of professional and personal reasons. Because such Investigators will have to be replaced, the new Investigator will assume the masthead authorship role for the Trial site. Ordinarily, the role of such Investigators can be acknowledged in an appendix. For some studies, for example ancillary studies in which the former Investigator may have played a primary or very strategic role, the Publications

Committee may elect to recognize the important contribution of the former Investigator by including him/her as an author (even to appoint him/her to a Writing Group). Similarly, the Publications Committee would be the entity that would entertain a petition from the former Investigator to be included as an author. If the former Investigator meets the criteria for authorship, as defined above, the Publications Committee will have the discretion to include him/her as an author. If the former Investigator's petition is denied by the Publications Committee, the Investigator will have recourse to a final decision by the Steering Committee. Similarly, if Publications Committee deliberations fail to resolve a dispute over authorship of a former Investigator, the dispute will be submitted for resolution to the Steering Committee.

9. Non-HALT-C Trial Investigators

a. For some ancillary studies, HALT-C Trial investigators may wish to invite outside experts to participate as scientific collaborators. Such addition of investigators should be presented for approval to the Ancillary Studies Committee, and this approval should be ratified by the Steering Committee. Ordinarily, manuscript authorship would be limited to HALT-C Trial investigators. If, however, such an outside expert has played a primary or very strategic role in the study, the Publications Committee may elect to recognize the important contribution of the outside Investigator by including 5him/her as an author (even to appoint him/her to a Writing Group). Such a request should be presented by a HALT-C Trial member of that ancillary study. If the outside investigator meets the criteria for authorship, as defined above, the Publications Committee will have the discretion to include him/her as an author. If the petition from the HALT-C Investigator to include the outside Investigator as an author is denied by the Publications Committee, the HALT-C Investigator making the proposal will have recourse to a final decision by the Steering Committee. Similarly, if Publications Committee deliberations fail to resolve a dispute over authorship of an “outside” investigator, the dispute will be submitted for resolution to the Steering Committee.

E10. Ancillary Study Investigators Who Are Not Members of the Steering Committee

a. Several ancillary studies involve internal collaborators who are not part of the Steering Committee. Such investigator groups will follow the HALT-C Publication and Presentation Guidelines. Within the ancillary study group, however, more specific guidelines may need to be developed. The object of such ancillary-study-group-specific guidelines will be to define the conduct of Writing Groups, determine Order of Authorship within the group, and establish timelines for receipt by the Publications Committee of documents and slide material to meet publication, abstract submission, and presentation deadlines.

F. SELECTION OF TOPICS

1. The main outcome report shall address the impact of long-term antiviral therapy on precirrhotic and cirrhotic chronic hepatitis C, i.e., the primary objective of the HALT-C Trial.

2. Another obvious outcome to be addressed in a published report will be the response of previous interferon nonresponders to "lead-in" therapy with pegylated interferon plus ribavirin.

3. The Publications Committee may suggest topics to be covered in manuscripts, and these will be discussed during Publications Committee meetings and conference calls.

4. Record of topics for other manuscripts will be kept by the Publications Committee and expanded as the study progresses.

5. Any member of the Steering Committee can propose a topic for an abstract or manuscript (other investigators should channel their requests through the Steering Committee member of their study site). Proposals should be submitted in writing to the Publications Committee; a summary of up to one page should include (on a form to be designed by the Data Coordinating Center)

   a. a brief description of the background/hypothesis/purpose
   b. a definition of the subjects to be included
   c. a list of variables of interest
   d. a list of possible collaborators
   e. for abstracts, the date of submission and date of the meeting

6. Criteria for judging proposals to the Publications Committee:

   a. scientific merit of the hypothesis or aim of the proposal
   b. availability of appropriate data to address the hypothesis or aim

7. Overlap between proposals: If overlap in content exists between two proposals, the chairperson of the Publications Committee will arrange a conference call among the two investigators, the chairperson of the Publications Committee, and the chairperson of the Steering Committee to review the two proposals and either eliminate overlap or consolidate the two proposals. Alternatively, the Publications Committee will make these determinations, based upon principles outlined in section C.3, above.

8. For approved proposals, the Publications Committee will ask the Data Coordinating Center to distribute a copy to all Principal Investigators (members of the Steering Committee). Within three weeks of receiving the proposal, each participating institution will forward to the Chair of the Publications Committee a list of investigators who would like to participate in the Writing Group. The Publications Committee will then select at least four to five investigators from among the proposed participants (a larger number at the discretion of the

Publications Committee) to constitute the Writing Group and appoint a member of the Writing Group to chair the group; this person will usually be the person who proposed the project. As is the case for Main Papers, the selection of Writing Groups and their chairpersons will be submitted to the Steering Committee for final approval.

G. RESPONSIBILITIES OF THE WRITING GROUPS

1. The Writing Groups are responsible for development of the full proposal for final approval by the Publications Committee. The full proposal should be submitted to the Publications Committee and should include the following:

   a. the basic analytic approach (preliminary, univariate, multivariate analyses)
   b. mock tables to include which variables are involved at each stage and in what combination
   c. for multivariable analysis, the description of the model, including dependent and independent variables
   d. the graphic needs of the final manuscript
   e. target audience and potential journal
   f. proposed timeline for each stage of the analysis and writing
   g. plans for meeting with the statisticians at the Data Coordinating Center
   h. proposed first author and list of co-authors

2. All data analysis will be done through the Data Coordinating Center, which will evaluate requests for the items listed above (G.1. a-d, g). Based upon their review of these requests, the DCC should provide an estimate of the time and resources required. A preliminary time-and-resource estimate should be included in the proposal of the Writing Group after consultation with the DCC.

3. The Chairperson of the Writing Group will be responsible for assigning tasks to other members of the Writing Group and for overseeing the completion of these tasks on schedule. Writing Group members should participate actively in the writing and review of the manuscript assigned to the Group.

4. If, during the course of work on a manuscript, the analysis is found to be too broad for a single manuscript, the Writing Group may suggest that the data would be more suitable for more than a single manuscript. The Writing Group should notify the Publications Committee that they plan to narrow the scope of the manuscript, and/or they can resubmit a new written plan to the Publications Committee for other potential manuscripts. An amended analysis plan should be submitted to the Publications Committee, if the analysis evolves or deviates substantially from that in the original plan filed with the Publications Committee.

5. Manuscripts should be prepared at the center of the Writing Group Chairperson, with assistance from the Data Coordinating Center. The completed manuscript should be submitted for review to members of the Publications Committee and NIDDK for final review.

H. ADMINISTRATIVE DETAILS

1. All manuscripts and abstracts should include “the HALT-C Trial Group” in the list of authors.

2. Each manuscript emanating from the HALT-C Trial will be numbered, and the number should be included in a footnote or acknowledgment on the title page of the manuscript as follows: “This is publication 1 of the HALT-C Trial.” Such numbering will apply to full manuscripts, not to abstracts.

3. All HALT-C Trial manuscripts should include an acknowledgment of NIDDK funding, with specific contract numbers (all the 11 contract numbers are needed), as well as NIH funding numbers of participating General Clinical Research Centers. Acknowledgment of partial funding by Hoffmann-La Roche should be included as well. When appropriate, other-institute support should be acknowledged, for example, NIAID support for publications emanating from the Virology/Immunology Ancillary Study; other support to be cited would include the National Cancer Institute, National Center for Minority Health and Health Disparities, etc., as appropriate for an individual manuscript. For abstracts, acknowledgments can be limited to NIDDK funding without fund numbers, which would be too numerous to list.

4. Requests for reprints of Main Papers, other study-wide manuscripts, and local papers can be addressed to, and distributed by, either the DCC or the First Author, at the First Author’s discretion.

5. For each HALT-C Trial manuscript, a paragraph that contains financial disclosures for all authors should be submitted. The financial disclosure should be incorporated into the text of the manuscript or into the letter of submission, according to the convention of the specific journal. This financial disclosure will be based on the information that the DCC has collected from the authors.

I. EDITORIAL FUNCTIONS

1. The Publications Committee will serve as the editorial review committee for all manuscripts.

2. For each manuscript prepared, two Publications Committee members who have not written the paper will be designated to provide a timely review (within three weeks) of the manuscript for editorial clarity and data integrity before the manuscript is submitted for publication.

3. The Publications Committee may suggest modifications before final approval or may suggest alternative journals.

4. Manuscripts, abstracts, or other publications that involve study-drug treatment or that focus on virologic or other test results obtained with commercial assays should be submitted to the industrial sponsor for comment at the same time as submission to NIDDK; however, the industrial sponsor will have no authority to prevent or delay publication.

5. If a dispute occurs between the authors and the Publications Committee, resolution of the dispute should be the responsibility of the Steering Committee.

J. ABSTRACTS AND PRESENTATIONS

1. Proposals for submission of abstracts should be submitted to the Publications Committee at least 6 weeks prior to the submission deadline. Priority for data analysis will be given to abstracts by the Publications Committee in consultation with the Data Coordinating Center. Assignments may be made to already existing Writing Groups, or a new Writing Group may be formed according to guidelines listed above. The complete proposal for an abstract may be briefer than one for a manuscript, but accepted abstracts require a complete proposal submission to the Publications Committee.

2. Abstracts should be submitted to the Publications Committee at least 7 days prior to submission to the organization sponsoring the meeting. The Data Coordinating Center will distribute the abstract to all Steering Committee members for review, and two members of the Publications Committee will be selected by the Chair of the Publications Committee for expedited review.

3. Abstracts will also be submitted to the NIDDK at least 7 days prior to submission.

4. Slide material (including tables and graphs) to be presented for accepted abstracts and posters should be reviewed by the Publications Committee at least 7 days prior to presentation.

5. Unpublished HALT-C Trial data presented at national and international meetings must be approved in the same way as abstracts. This may entail review of slides and printed material by the same mechanism as that used to review abstracts. When previously approved slides are to be presented at a national or international meeting, the Publications Committee should be notified at least 7 days in advance, the slides should contain an acknowledgment of the original source, but formal re-approval will not be required. Requests for permission by meeting organizers, industrial sponsors, or non-HALT-C investigators to reproduce slides, to videotape or audiotape presentations, and/or to publish written summaries of these presentations must be submitted to the Publications Committee, which will review the material to ensure that previously unpublished data are protected. Publications Committee approval is not required for local presentations and accompanying syllabus material (medical school lectures, continuing education courses, grand rounds lectures, research seminars, etc.). Investigators are encouraged to consult

the Publications Committee chairperson when questions about the propriety of a local presentation arise. If the chairperson cannot address such questions readily, the issue should be considered by the entire Publications Committee (via conference call or written communication).

6. Abstract citation of support and HALT-C Trial label:

   a. With the first mention of HALT-C in an abstract, the name should be spelled out [Hepatitis C Antiviral Long-Term Treatment against Cirrhosis (HALT-C) Trial]. This should be in the title or in the body of the abstract. Also, as indicated in H.1., above, all manuscripts and abstracts should include "…and the HALT-C Trial Group" in the list of authors in the authorship line.

   b. Suggested abstract funding acknowledgment: "Funded by the National Institute of Diabetes and Digestive and Kidney Diseases (NIDDK for brevity, if needed), with additional support from others as appropriate for an individual abstract, such as NIAID for immunology studies.

   c. In any slide presentation or poster, including presentations of data at HALT-C Trial Steering Committee meetings, if the presenter chooses to display a logo, the primary logo should be that of the HALT-C Trial. The presenter may choose as well to display the logo of his/her institution.

7. Letters to the Editor should be approved according to the same process as that used for abstracts.

K. NIDDK APPROVAL

1. Final manuscripts prepared by members of the Writing Groups and Publications Committee should be approved by the NIDDK before submission for publication. The NIDDK should complete its review within 30 days, after which, if no objections are raised, the manuscript can be submitted. (For abstracts, submission to the NIDDK will be required 7 days prior to submission.) (Although approval by the industrial source of study medications is not required, as a courtesy, manuscripts that include drug treatment or that focus on virologic or other test results obtained with commercial assays will be submitted to the industrial sponsor at the same time as the NIDDK. As noted above, the industrial sponsor will not have authority to prevent or delay publication.)

L. PUBLICATION PRIORITIES AND ACCESS

1. No investigator should jeopardize the publication of HALT-C Trial data in a peer-reviewed journal by releasing or presenting data prematurely. Press releases should be timed to coincide with publication of manuscripts and should respect any applicable publication embargoes.

2. No individual site will be permitted to publish site-specific results that compete and interfere with the integrity of study-wide reports of the results of the HALT-C Trial.

3. Press Releases should be approved by the Publications Committee and the Steering Committee.

4. All manuscripts and abstracts will be stored electronically on the HALT-C Trial secure website, to which all study personnel have access. Slide material prepared for presentations should be made available to other HALT-C investigators in electronic format, to be distributed as an e-mail attachment by the Data Coordinating Center.

Acknowledgments

In drafting these publications guidelines for the HALT-C Trial, we had the benefit of referring to publications guidelines from the following sources: authorship guidelines of the Annals of Internal Medicine and publications guidelines of the Hepatitis Interventional Therapy Group; the NIAID-sponsored NCICAS (ACTIVE Study); the NHLBI-sponsored SHOCK Trial and Registry; the NIA-sponsored Health, Aging, and Body Composition Study (HEALTH ABC); and the NHLBI-sponsored Natural History of Transfusion-Associated Non-A, Non-B Hepatitis Study.
