# Supplementary material for: Processes to manage analyses and publications in a phase III multicenter randomized clinical trial
Source: Trials. 2014 May 7;15:159. doi: 10.1186/1745-6215-15-159 (PMC4040510; doi:10.1186/1745-6215-15-159)
Supplement: Additional file 3 — HALT-C Trial Data Use Agreement. Data Use Agreement template. [file 1745-6215-15-159-S3.pdf]

Hepatitis C Antiviral Long-term Treatment against Cirrhosis

HALT-C Trial
Data Coordinating Center

HALT-C Trial Data Use Agreement

Date:

The Hepatitis C Antiviral Long-term Treatment against Hepatitis C (HALT-C) Data Coordinating Center (DCC), located at New England Research Institutes, 9 Galen Street, Watertown, MA 02472 will provide the investigator below (Recipient) with de-identified patient-level data (Data) described below extracted from the HALT-C research database maintained by the HALT-C DCC, solely for the Use identified below.

Recipient's Name, Institution, Street Address, Telephone Number, Contact Name, Contact Telephone Number, Email:

Description of Data to be Delivered, Date of Delivery, and Format:

Purpose for Which Recipient Desires to Use Data (Attach Research Plan):

Assurance of Confidentiality of Data (Attach Security Plan)

Approval by HALT-C Steering Committee (Attach copy of Letter of Approval)

The entire Data Use Agreement incorporates this document, agreement clauses, research plan, and security plan.

AGREED AND ACCEPTED:

____

Recipient typed name **Title**

____

Recipient signature **Date**

____

DCC Principal Investigator **Date**

____

NIDDK Project Officer **Date**

HALT-C Data Use Agreement Attachment – Agreement Clauses

The purpose of this agreement is to maintain the confidentiality of patient data. Data distributed under this agreement will have been stripped of all identifiers. Patient IDs will be replaced with new randomly generated ID numbers. Site identifiers, if needed, will also be randomly assigned. All dates will be removed from the data set. Age at entry will be used instead of date of birth and all other dates will be expressed as weeks or months since entry. Despite this, it might be possible to identify individual subjects. The recipient must therefore adhere to the requirements of this data use agreement.

The Recipient acknowledges, agrees, and represents that, unless otherwise expressly permitted in writing by the HALT-C DCC:

1. It shall not make copies of the Data, and shall not sell information derived from the Data.

2. It shall not use the Data to identify individuals, and will not link or combine the Data with other information for the purpose of identifying individuals.

3. The HALT-C Steering Committee must approve the proposed study. These data may not be linked to other data sets without prior justification and approval by the DCC and Steering Committee, consistent with the research plan.

4. It shall use the Data solely for bona fide research/analysis described in the Purpose set forth above, and specifically shall not use the Data for any commercial purpose which could have a negative impact on patient welfare, such as offering, denying, or allocating insurance; and adverse selection (*e.g.*, identifying patients with high risk diagnoses).

5. It shall not publish or otherwise disclose the Data to any person or organization unless the Data shall have been aggregated (that is, combined into groupings of data such that the data are no longer specific to any individuals within each grouping), <u>and</u> the nature and purpose for such disclosure is set forth in the authorized Use, above.

6. It shall not publish or otherwise disclose Data that permits the identification of individual providers or facilities; provided, however, the Recipient may release Data to a contractor for purposes of data processing or storage if (1) the Recipient specifies in the research plan submitted to the HALT-C DCC that data would be released to the particular contractor, or the Recipient has obtained written authorization from the HALT-C DCC to release the data to such contractor, <u>and</u> (2) the contractor has signed a data release agreement with the HALT-C DCC.

7. It shall provide to the HALT-C DCC a copy of any aggregation of Data intended for publication for review for compliance with the terms of this agreement and with the terms of the HALT-C Publications and Presentations Guidelines prior to submission for publication and, if not approved, shall not be published. The HALT-C DCC shall respond to any such submission within thirty (30) days of receipt.

8. It acknowledges that the Data are private and confidential, and that unauthorized use is a violation of the terms of this Agreement and may subject the Recipient and its employees to legal sanctions and termination of use of the Data.

9. It has in place, and shall maintain throughout the proposed study, administrative, technical, procedural, and physical safeguards sufficient to protect the confidentiality of the Data and to prevent unauthorized access and use. The safeguards shall provide a level of security outlined in OMB Circular No. A-130, Appendix III — Security of Federal Automated Information System, which sets forth guidelines for security plans for automated information systems in Federal agencies.

10. At the completion of the project, it shall return the Data, or shall certify in writing the deletion and destruction of all copies of the Data.

11. Only those employees who have a “need to know” shall access the Data, and all such employees shall be advised of the terms of this Agreement and the restrictions upon use and disclosure.

12. It shall keep an accurate written account of all authorized copies of the Data, and of work product derived from the Data, and shall furnish such written logs upon request to the HALT-C DCC.

13. It shall permit authorized representatives of the HALT-C DCC and/or NIDDK access to premises where Data are kept for the purpose of inspecting security procedures and compliance with the terms of this Agreement.

14. The HALT-C DCC represents that the release of data described in this Agreement does not violate applicable laws or contractual requirements, including the Privacy Act, the Freedom of Information Act, the Trade Secrets Act, and Federal and State laws on the confidentiality of patient records or trade secrets.

HALT-C Data Use Agreement Security Plan Questions

Name of Researcher____Date_______________

1. On what type of computer system will the data be stored and used? Please answer this for each computer on which the data will be stored or used. Check all that apply.

   ☐ On one or more laptops
   ☐ On one or more desktop PCs or other non-portable single-user computers
   ☐ On one or more PCs connected to a LAN with data on a network file server
   Please describe any other arrangement.

2. What operating system(s) will be used?

3. Who will have access to the data? (Per DUA item 11, "Only those employees who have a 'need to know' shall access the Data, and all such employees shall be advised of the terms of this Agreement and the restrictions upon use and disclosure.") List their names and titles.

4. How will access to the data be limited to these authorized persons? Include both electronic and physical controls, where applicable.

5. What is the security plan for this computer system providing for protection of patient-specific data? Please provide a brief description.

6. What virus protection software, including version number, is used on this system? When was it last updated (usually found through the Help/About menu)? How often is the virus protection software updated?

7. What spy ware protection software, including version number, is used on this system? When was it last updated (usually found through the Help/About menu)? How often is the spy ware protection software updated?

8. Does each computer automatically lock, requiring a password to unlock, after some time interval of inactivity? What is the time interval?

9. How is the computer physically secured when not in use? Is there at least one locked door between the computer and the public when it is not in use?

10. If one or more laptops are used, how is the hard drive encrypted (specify the software product and version)? If not, how are all files with patient-specific data protected on the laptop?

11. If you are connected to a LAN/Internet, are you behind a firewall?

12. How and onto what media will backup copies of the data be made? Where will the original CDs or other media from the HALT-C and these backup copies be stored?

13. How will you keep an accurate written account of all authorized copies of the Data, and of work product derived from the Data (per DUA item 12) so that, at the completion of the project, you will be able to return the Data, or shall certify in writing the deletion and destruction of all copies of the Data (per DUA item 10)?

14. Where will printouts of patient-specific data be stored when not in use?

15. How are users trained in the rules governing the data and the computer system and in the procedures to be followed? (per DUA item 11, "Only those employees who have a 'need to know' shall access the Data, and all such employees shall be advised of the terms of this Agreement and the restrictions upon use and disclosure.")

The HALT-C DCC may ask for additional information to clarify the answers provided above.

Notes on Completion of HALT-C Data Release Agreement Security Plan

In item 9 in the HALT-C Data Use Agreement (DUA) clauses, the recipient represents that:

> "…it has in place, and shall maintain throughout the proposed study, administrative, technical, procedural, and physical safeguards sufficient to protect the confidentiality of the Data and to prevent unauthorized access and use. The safeguards shall provide a level of security outlined in OMB Circular No. A-130, Appendix III — Security of Federal Automated Information System, which sets forth guidelines for security plans for automated information systems in Federal agencies."

Appendix III to OMB Circular No. A-130 (OMB A-130) can be found at the following website: http://www.whitehouse.gov/omb/circulars/a130/a130trans4.html. The National Institute of Standards and Technology (NIST) publication 800-18 provides detailed standards for information system security plans. OMB A-130 recognizes that the security plan should be cost-effective and commensurate with risk. It defines "adequate security" as "… security commensurate with the risk and magnitude of the harm resulting from the loss, misuse, or unauthorized access to or modification of information. This includes assuring that systems and applications used by the agency operate effectively and provide appropriate confidentiality, integrity, and availability, through the use of cost-effective management, personnel, operational, and technical controls."

The primary harm that may be involved with release of HALT-C data to researchers is unauthorized release of confidential and potentially identifiable patient data. HALT-C data releases do not include patient identifiers but are "patient identifiable" in the sense that patients might be identified using other data or information in combination. Note that while OMB A-130 is concerned with integrity and availability and well as confidentiality, HALT-C's principal concern is confidentiality of the data.

For HALT-C data releases, the researcher is responsible for security, the security plan is part of the data release process, the time horizon does not require periodic review, and the Data Use Agreement provides authorization.

When submitting the Data Use Agreement, please attach either answers to these specific questions, or an existing organizational security plan that addresses these questions. In the case of the latter, specific reference to each question may expedite the security approval process
